# Expression of Concern to: Redox modification of cysteine residues regulates the cytokine activity of high mobility group box-1 (HMGB1)

**DOI:** 10.1186/s10020-020-0144-8

**Published:** 2020-02-04

**Authors:** Huan Yang, Peter Lundbäck, Lars Ottosson, Helena Erlandsson-Harris, Emilie Venereau, Marco E. Bianchi, Yousef Al-Abed, Ulf Andersson, Kevin J. Tracey, Daniel J. Antoine

**Affiliations:** 1 Laboratory of Biomedical Science, The Feinstein Institute for Medical Research, Manhasset, New York, USA; 2 Departments of Women’s and Children’s Health, Medicine and Rheumatology Research Laboratory, Karolinska Institutet and Karolinska University Hospital, Stockholm, Sweden; 3 San Raffaele University and Scientific Institute, Milan, Italy; 4 Department of Medicinal Chemistry, The Feinstein Institute for Medical Research, Manhasset, New York, USA; 5 MRC Centre for Drug Safety Science, Department of Molecular and Clinical Pharmacology, University of Liverpool, Liverpool, UK

**Expression of Concern to: Mol Med (2012) 18:250–259**

**https://doi.org/10.2119/molmed.2011.00389**

The Editors-in-Chief would like to alert readers that this article (Yang et al. 2012) is part of an investigation being conducted by the journal following the conclusions of an institutional enquiry at the University of Liverpool with respect to the quantitative mass spectrometry-generated results regarding acetylated and redox-modified HMGB1. Appropriate editorial action will be taken once the investigation is concluded.

Huan Yang, Peter Lundbäck, Lars Ottosson, Helena Erlandsson-Harris, Emilie Venereau, Marco E. Bianchi, Yousef Al-Abed, Ulf Andersson, and Kevin J. Tracey agree to this editorial expression of concern.

Daniel J. Antoine has not responded to any correspondence from the editor/publisher about this editorial expression of concern.
